# Supplementary material for: Newborn Screening for Severe Combined Immunodeficiency: Lessons Learned from Screening and Follow-Up of the Preterm Newborn Population
Source: Int J Neonatal Screen. 2023 Dec 15;9(4):68. doi: 10.3390/ijns9040068 (PMC10744167; doi:10.3390/ijns9040068)
Supplement: Supplementary file 1 [file IJNS-09-00068-s001.zip › Supplementary Document S1.pdf]

EXCLUSION CRITERIA, COHORTS, AND DATA DEFINITION MATRIX FOR SCID PREMATURITY QI PROJECT

EXCLUSION CRITERIA
**BABIES WITH BIRTHWEIGHT >2500 grams**
**BABIES WITH GESTATIONAL AGE ≥37 weeks**
**UNSATISFACTORY SPECIMENS; INCLUDING BUT NOT LIMITED TO:**<br>• Poor Quality<br>• Transit Issues<br>• No DNA Amplification<br>• Any other reason(s) your laboratory may classify a specimen as unsatisfactory or invalid
**BABIES WITH OUT-OF-RANGE SCREENING RESULTS FOR ANYTHING OTHER THAN SCID/T-CELL LYMPHOPENIA (TCL)**

*** NOTE: If both birthweight and gestational age are collected and do not both fall in the premature/LBW category, please use gestational age as the deciding factor (e.g., a newborn with a birthweight of 2550g and a GA of 35 weeks would be *included*. A newborn with a birthweight of 2300g and a GA of 38 weeks would be *excluded*).**

COHORT	DESCRIPTION
**Random Sampling** of Low Birth Weight/Premature Babies with **ALL WITHIN-RANGE (SCREEN-NEGATIVE) Newborn Screening Results**	• Only include babies with WITHIN-RANGE newborn screening results for ALL disorders<br>• Exclude any infants with A>F (transfusion) results<br>• Only provide information from the initial valid screen collected between **24-48 hours of age**
**ALL** Low Birth Weight/Premature Babies with **OUT-OF-RANGE SCID/TCL Newborn Screening Results**	• Include any babies who had an OUT-OF-RANGE (repeat screen or clinical evaluation request) for SCID/TCL<br>• Include information on ALL associated screens for the patient

RANDOM SAMPLING INSTRUCTIONS

1. For each calendar year requested, you will provide APHL with a data file that only contains the following elements:
   a. State Abbreviation (e.g, NY, MN, etc)
   b. Patient Identifier
   c. Birthweight
   d. Sex
   e. Gestational Age
   f. Birth year

2. From this file, we will apply our own process to get a random sampling and send you back a list of patient identifiers for which we would like the additional related information
   a. You will then take that list of patients, and send us back the complete information as outlined below

DATA DEFINITION MATRIX FOR PATIENT LEVEL DATA

FIELD NAME	DATA TYPE	DATA FORMAT	DESCRIPTION	EXAMPLE	REQUIRED FOR?	ACCEPTABLE NULL VALUE?
**State**	Char	XX	Two Letter State Abbreviation	NY	Screen-Negative Cohort<br>Out-of-Range SCID Cohort	N
**Patient ID**	VarChar		Unique Patient Identifier		Screen-Negative Cohort<br>Out-of-Range SCID Cohort	N

			Same Patient Identifier should be repeated for all screens associated with a single patient<br><br>Patient Identifier can be Program Defined			
**Birthweight**	Numeric	NNNN	Birthweight in Grams	1800	Screen-Negative Cohort<br>Out-of-Range SCID Cohort	N
**Gestational Age**	Numeric	NN	Gestational Age (in weeks truncated to the integer portion – ex. 35 and 6 days would become 35 weeks gestation)	32	Screen-Negative Cohort<br>Out-of-Range SCID Cohort	Y
**Birth Year**	Date	YYYY	Year of Birth	2019	Screen-Negative Cohort<br>Out-of-Range SCID Cohort	N
**Sex**	Char	X	Indicate reported sex of newborn at birth:<br><br>M = Male<br>F = Female<br>U = Unknown	F	Screen-Negative Cohort<br>Out-of-Range SCID Cohort	N
**Age at Time of Collection**	Numeric	NN	Age at Collection in Hours	36	Screen-Negative Cohort<br>Out-of-Range SCID Cohort	N
**TREC/Ct/MoM Value**	Numeric		Provide numeric value for TREC/Ct/MoM values.<br><br>If have multiple values, please only provide the value that is used to determine the screening results/interpretation in your program		Screen-Negative Cohort<br>Out-of-Range SCID Cohort	N

			Provide results to whichever decimal place you use in your program			
**Analyte Value Units**	VarChar		Use the Following Descriptors Only:<br>• **TRECs/µl**<br>• **Ct (or Cq)**<br>• **MoM**		Screen-Negative Cohort<br>Out-of-Range SCID Cohort	N
**Median TREC or Ct Value *(*for those reporting TRECs/µl or Ct values only)***	Numeric		Provide the population median used to calculate the cut-off utilized at the time the newborn was screened<br><br>***NOTE**: This value will likely be the same across multiple newborns and will only change if the cut-off was changed secondary to a shift in population median*		Screen-Negative Cohort<br>Out-of-Range SCID Cohort	N
**Transfusion Status**	Y/N/U	X	Indicate whether the child was transfused (using results from the Hgb assay indicating A>F) as:<br><br>**Y: Yes**<br>**N: No**<br>**U: Unknown**<br><br>Use 'U' in the following circumstances:<br>• Discrepancy is seen between information provided on NBS kit and Hgb assay results	Y	Out-of-Range SCID Cohort	N

			• A>F findings in older babies who may have already transitioned from fetal to adult hemogloblin			
**Screen Interpretation/ Resulting Action**	VarChar		Use the Following Descriptors Only:<br><br>• **Additional Repeat Requested** *(Only includes additional requested repeat screens outside of the normal, routine screening process)*<br><br>• **Routine Repeat Process** *(Use this if the recommendation is to simply follow the typical screening process (e.g., for LBW infants, the recommendation is to wait for the normal second specimen)*<br><br>• **Clinical Evaluation Recommended**		Out-of-Range SCID Cohort	N
**Final Outcome**	VarChar		Use the following Descriptors Only:<br><br>• **SCID** *(Includes Typical, Omenn, Leaky)*<br>• **Non-SCID T-cell lymphopenia** *(Includes syndrome, secondary)*<br>• **Transient T-cell lymphopenia**<br>• **False positive** (*Use this designation only if the newborn had a diagnostic clinical work-up*)		Out-of-Range SCID Cohort	N

			• **Normal repeat screen**<br><br>Programs are highly encouraged to use NewSTEPs case definitions to report final outcome.			
**Other Relevant Demographic or Medical Information**	VarChar		If available, provide any additional relevant medical information that may help explain the SCID/TCL screening results. Examples include: mother receiving immunosuppressive therapy, cardiac surgery on baby, Plain clothes community, etc.		Out-of-Range SCID Cohort	Y
**Flow Data**	Var Char<br>Numeric		If available, provide the following values for confirmed True Positive cases:<br><br>• CD45RA<br>• CD3+<br>• ALC (absolute lymphocyte count)		Out-of-Range SCID Cohort	Y

AGGREGATE SCID/TCL DATA FOR YEARS 2018, 2019, AND 2020

DATA ELEMENT	DESCRIPTION
**Total number of babies screened by NBS Program**	Provide total number of babies screened (regardless of birthweight/GA) by each calendar year

	This number should be the total number of babies screened by the program, regardless of state of residence
**Total number of babies with out-of-range results for SCID/TCL**	Provide total number of babies (regardless of birthweight/GA) with out-of-range results for SCID/TCL by each calendar year
**Total number of babies with out-of-range SCID/TCL results where a repeat screen was requested**	Of the total number of babies (regardless of birthweight/GA) with out-of-range results for SCID/TCL, provide the number of babies whose results led to a request for a repeat NBS screen<br><br>***Only count a baby once. If a baby had both a 'repeat requested' result and a 'clinical evaluation' result, count the baby in the 'Total number of babies with out-of-range SCID/TCL results where clinical evaluation was recommended' category***
**Total number of babies with out-of-range SCID/TCL results where clinical evaluation was recommended**	Of the total number of babies (regardless of birthweight/GA) with out-of-range results for SCID/TCL, provide the number of babies whose results led to a recommendation for clinical evaluation<br><br>***Only count a baby once. If a baby had both a 'repeat requested' result and a 'clinical evaluation' result, count the baby in the 'Total number of babies with out-of-range SCID/TCL results where clinical evaluation was recommended' category***
