# Supplementary figures and images for: Newborn Screening for Severe Combined Immunodeficiency: Lessons Learned from Screening and Follow-Up of the Preterm Newborn Population
Source: Int J Neonatal Screen. 2023 Dec 15;9(4):68. doi: 10.3390/ijns9040068 (PMC10744167; doi:10.3390/ijns9040068)

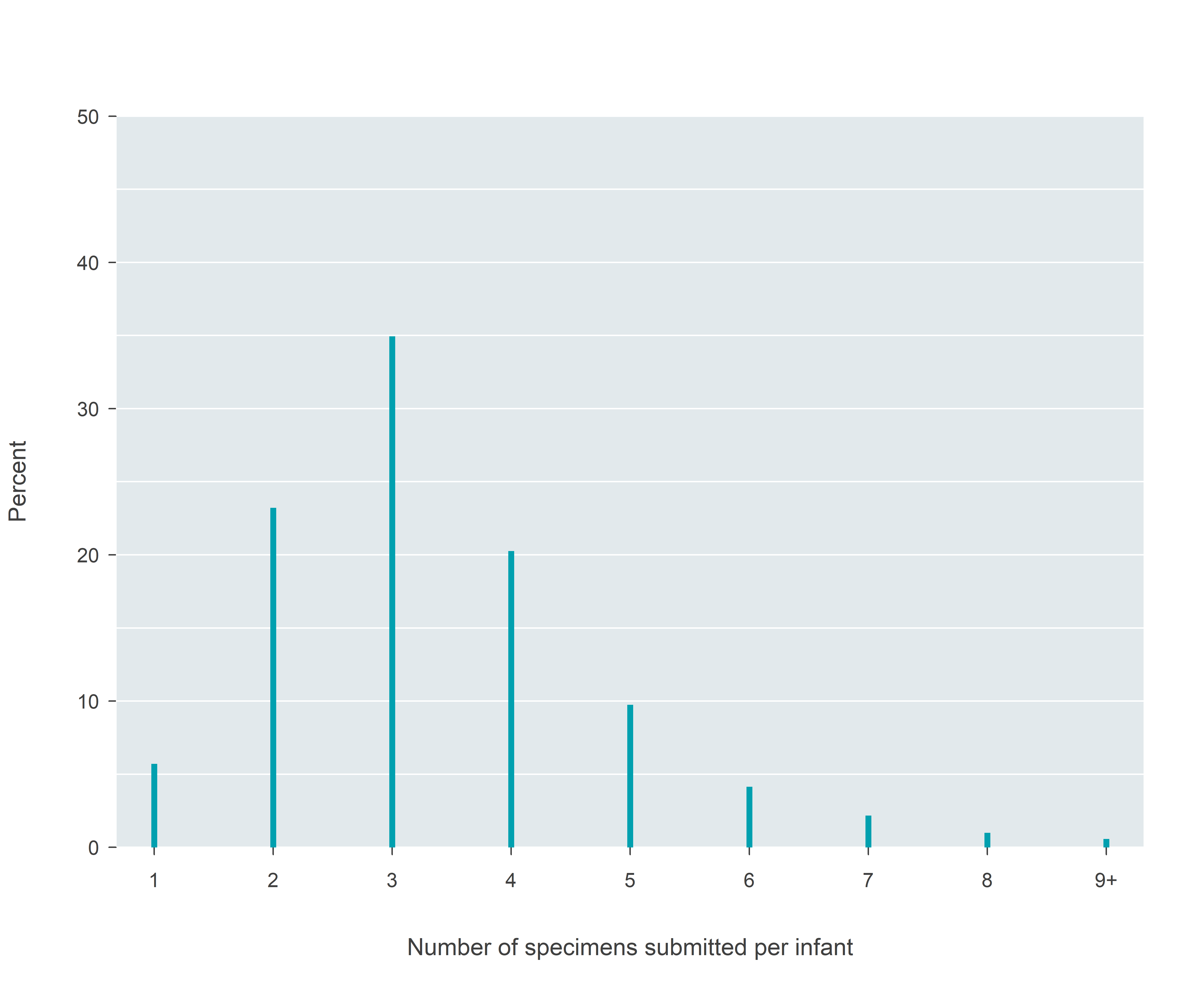

Supplement: Supplementary file 1 [file IJNS-09-00068-s001.zip › Figure S1.png]
